# Sediment and Salinity Thresholds Govern Natural Recruitment of Manila Clam in the Xiaoqing River Estuary: Toward a Predictive Management Framework

**DOI:** 10.3390/biology15020157

**Published:** 2026-01-15

**Authors:** Lulei Liu, Ang Li, Shoutuan Yu, Suyan Xue, Zirong Liu, Longzhen Liu, Ling Zhu, Jiaqi Li, Yuze Mao

**Affiliations:** 1 State Key Laboratory of Mariculture Biobreeding and Sustainable Goods, Yellow Sea Fisheries Research Institute, Chinese Academy of Fishery Sciences, Qingdao 266071, China; liull@ysfri.ac.cn (L.L.); lia@ysfri.ac.cn (A.L.); xuesy@ysfri.ac.cn (S.X.); m1727075341@163.com (Z.L.); llongzhen1990@163.com (L.L.); zhuling@ysfri.ac.cn (L.Z.); lijq@ysfri.ac.cn (J.L.); 2 Laboratory for Marine Ecology and Environmental Science, Qingdao Marine Science and Technology Center, Qingdao 266237, China; 3 Hongdao Subdistrict Office of Chengyang District in Qingdao City, Qingdao 266114, China; tuanshouyu@163.com

**Keywords:** *Ruditapes philippinarum*, environmental thresholds, sediment grain size, salinity stress, natural recruitment

## Abstract

Natural recruitment of Manila clam (*Ruditapes philippinarum*) often persists in degraded estuaries, yet the environmental thresholds enabling this resilience remain quantitatively undefined. We employed binomial generalized additive model (GAM) coupled with field surveys (*n* = 168) in the Xiaoqing River estuary (Laizhou Bay, China) to identify critical limits for adult occurrence, which served as a field-based proxy for recruitment potential. Sediment median grain size (D_50_), salinity (Sal) and dissolved inorganic nitrogen (DIN) were identified as the key factors, collectively explaining 79.30% of the deviance (AUC = 0.98). The probability of occurrence decreased sharply beyond two distinct thresholds: D_50_ > 95 μm and salinity < 17.50‰. While DIN had a positive effect, it did not offset the strong negative associations with coarse sediment or low salinity. These field-validated thresholds provide quantifiable criteria to guide habitat suitability mapping, activation of early-warning systems against salinity-driven mortality, and site prioritization for ecological restoration in the Xiaoqing River estuary. Our findings offer a framework for developing management strategies to support clam resilience under environmental stress.

## Simple Summary

In some degraded coastal areas, Manila clams continue to survive and reproduce naturally, but it was unclear what specific environmental conditions allow this. Our study aimed to find these key conditions. We surveyed the Xiaoqing estuary and analyzed how clam presence relates to their habitat. We found two strict limits: clams almost disappear where the sediment particles become coarser than 95 μm or where the water salinity drops below 17.50‰. While nutrients in the water had a minor positive effect, they could not overcome poor sediment or low salinity. These clear thresholds provide science-based tools for managers: to map and prioritize areas for clam habitat restoration, and to create an early-warning system that alerts to low-salinity events which risk mass clam mortality. This work helps protect this ecologically and economically important shellfish in our changing coasts.

## 1. Introduction

Sustainable management of the Manila clam (*Ruditapes philippinarum*), an ecologically and economically vital bivalve along China’s coast, faces challenges from habitat degradation and climate-induced salinity fluctuations [1,2]. Yet, natural recruitment of marine benthos in degraded estuaries often exhibits unexpected resilience. Despite intense anthropogenic pressures, these ecosystems often retain a capacity for self-replenishment, as the Manila clam exemplifies. Widespread habitat deterioration and overexploitation have depleted natural stocks and eroded germplasm quality [3,4]; nevertheless, substantial spat recruitment occurs annually in Laizhou Bay. This suggests that microhabitat-scale environmental controls may override broader regional degradation. The potential for microhabitats to act as refugia aligns with general ecological principles. For marine bivalves, key factors include: (1) the persistence of a stable sedimentary template despite water-column perturbations [5]; (2) the buffering of acute salinity stress by local geomorphic features (e.g., deeper water layers) [6]; and (3) the existence of species-specific physiological thresholds that permit survival even when regional conditions deteriorate [7]. While this conceptual framework is well-established, the quantitative thresholds of sediment grain size and salinity that define functional refugia specifically for *R. philippinarum* remain critically undefined. Therefore, determining these specific thresholds is crucial to provide a quantitative basis for prioritizing protection efforts and designing effective habitat restoration.

Sediment grain size and salinity constitute primary determinants of bivalve distribution, as they regulate burrowing success, physiological performance, and survival [8,9]. Although previous studies have documented broad habitat preferences of *R. philippinarum* [10,11], critical gaps hinder predictive, resilience-based management. First, most research derives from aquaculture settings, leaving natural recruitment dynamics under sharp salinity gradients largely unexplored. Second, existing knowledge is primarily correlative and fails to identify the nonlinear threshold effects essential for predictive management. Most critically, there is no field-validated, quantitative salinity tolerance limit for episodic freshwater pulses—a growing threat under climate change. This absence prevents rigorous risk assessment and early warning for mass mortality events. Therefore, a study is needed to quantitatively define these critical thresholds in a natural estuarine setting.

To achieve this quantitative definition, we required a method capable of detecting complex, non-linear relationships without a priori assumptions. We therefore employed a Generalized additive model (GAM), which use smoothing splines to flexibly capture species-environment responses and visualize potential thresholds [12,13]. This approach quantifies the relative effects of multiple predictors simultaneously, overcoming the limitations of traditional linear regression for ecological threshold detection. While powerful in benthic assessment [14] and fisheries science [15,16], GAM is underutilized for identifying thresholds for natural *R. philippinarum* populations in dynamic estuaries. we applied binomial GAM to data from four surveys (*n* = 168) conducted across the Xiaoqing River estuary. This site provides a natural experiment, with its pronounced salinity fluctuations (16.99–21.92‰) and stable sedimentary conditions allowing us to disentangle substrate from osmotic controls.

This study aimed to quantify the sediment and salinity thresholds governing adult Manila clam occurrence. Given the technical challenges of directly monitoring larval and juvenile stages in dynamic estuaries, we used adult occurrence as a field proxy for recruitment potential. This proxy reflects integrated habitat suitability across life stages (see Section 4 for limitations). We tested the specific hypothesis that natural recruitment is sustained in only areas where sediment median grain size (D_50_) is below 95 μm and salinity exceeds 17.50‰. These threshold values were set as testable hypotheses based on (1) patterns from preliminary surveys in the study area, (2) the documented optimal sediment range for this species (50–100 μm), and (3) the known sensitive salinity zone (15–20‰) for venerid clams [11,17]. They were then rigorously evaluated using our modeling framework. Our study objectives were to: (1) quantify the relative contributions of sediment, salinity, and nutrients to adult occurrence; (2) identify the critical thresholds triggering abrupt distribution shifts; and (3) provide actionable criteria for restoration site selection and early-warning systems against salinity-driven mortality. By linking mechanism to management, this study provides a foundation for developing predictive strategies.

## 2. Materials and Methods

### 2.1. Sample Collection

**Figure 1:**
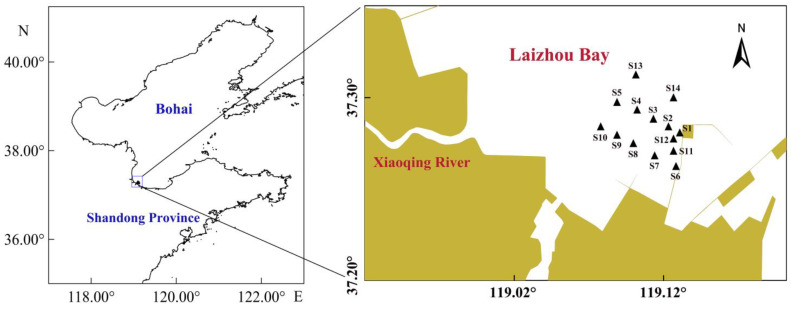
Location of the 14 sampling stations (S1–S14) in the Xiaoqing River Estuary, Laizhou Bay, China.

Four field surveys were conducted in 2024 (8 August, 22 August, 4 September, 24 September) to capture seasonal variations in salinity dynamics, particularly during the wet season when freshwater discharge peaks. The same set of fourteen fixed stations (S1–S14) was sampled during each survey in the Xiaoqing River estuary, Laizhou Bay (Figure 1). At each station, seawater temperature, salinity, and dissolved oxygen were measured in situ using a multiparameter water quality analyzer (Smartroll Mp, Fort Collins, CO, USA) [18]. Three 1 L surface water samples were collected per station in pre-labeled plastic containers. Sediment samples were obtained using a van Veen grab (0.1 m^2^) executed three times per station. In our design, the presence/absence of clams was recorded for each of the three replicate grabs collected at each station during each survey. We then manually separated Manila clams from the sediment. All clam and sediment samples were immediately stored at 4–8 °C for transport to the laboratory.

This study employed a fixed-station, repeated-measures design across a natural salinity gradient. This approach provided intrinsic field controls: stations acted as their own temporal controls across surveys, while spatial comparisons between habitats of differing suitability were enabled.

### 2.2. Laboratory Analysis

The concentrations of ammonia nitrogen (NH_4_-N), nitrate nitrogen (NO_3_-N), nitrite nitrogen (NO_2_-N), and reactive phosphorus (PO_4_-P) in water were determined following standard methods [19], which include prescribed quality control measures such as the analysis of duplicates and blanks. Data quality was thereby ensured. Dissolved inorganic nitrogen (DIN) concentration was calculated as the sum of NH_4_-N, NO_3_-N, and NO_2_-N. For chlorophyll-a (Chl-a) analysis, water samples were extracted with 10 mL of 90% acetone solution (*v*/*v*) for 24 h and then measured with a laboratory fluorometer (Trilogy, Turner Designs, San Jose, CA, USA) [20]. Prior to analysis, sediment samples were homogenized. An appropriate aliquot was placed in a beaker with approximately 15 mL of distilled water and stirred thoroughly. To remove organic matter and carbonates, 5 mL of 30% H_2_O_2_ and 5 mL of 10% HCl were added sequentially. After a 24 h reaction period, the supernatant was removed. Subsequently, 5 mL of sodium hexametaphosphate solution was added as a dispersant, and the mixture was subjected to ultrasonic oscillation to ensure complete dispersion [21]. The median particle diameter (D_50_) was then analyzed using a laser particle sizer (Mastersizer 3000, Malvern City, UK) [22]. The shell length and height of all collected clams were measured to the nearest 0.01 mm using digital calipers, and the total wet weight was determined to the nearest 0.01 g with an electronic balance.

### 2.3. Statistical Modeling

With the only assumption being that the relationship is additive and smooth [13], the generalized additive model (GAM) uses a link function to relate the conditional mean of the response variable to a set of smooth functions of the explanatory variables. The GAM is formulated as follows [14]:

(1) $$f\left(\mu \left(N\right)\right)=\beta_{0}+Y_{1}\left(x_{1}\right)+\ldots +Y_{n}\left(x_{n}+\varepsilon \right)$$

where *f*(.) is the connection function; *μ*(*N*) is the expected value of the response variable *N*; *β*_0_ is the intercept; and *Y*_i_(.) is the smoothing function for the *i*_th_ explanatory variable *x*_i_. ε is the residual.

### 2.4. Model Evaluation and Validation

The key assumptions of the binomial GAM were validated. The critical assumption of independent observations was upheld by our design (168 independent “station-survey” units) and confirmed by diagnostic tests on model residuals, which showed no significant spatial (Moran’s I: I = 0.08, *p* = 0.32) [23] or temporal autocorrelation (Durbin–Watson: statistic = 1.92, *p* = 0.38) [24]. Additionally, we conducted a robustness check by fitting a Generalized Additive Mixed Model (GAMM) [25] with random intercept for each Survey-Station combination to explicitly account for potential within-group correlation arising from repeated sampling.

The accuracy of the model fit was evaluated using the receiver operating characteristic (ROC) curve and the area under the ROC curve (AUC) [26]. The closer the curve approached the upper left corner, the higher the AUC value was, indicating better model performance. The AUC value ranged from 0 to 1, with values closer to 1 indicating better overall predictive performance of the model.

In this study, we defined the response variable (*Y*) as the presence (1) or absence (0) of *R. philippinarum* at each sampling station. This binary response was chosen because our data exhibited a high proportion of zeros, resulting in a zero-inflated distribution. Given our sample size, a binomial model is more statistically robust for such data than count-based alternatives (e.g., zero-inflated Poisson GAM) [27]. Each station-survey combination (14 stations × 4 × 3 surveys = 168 observations) was treated as an independent sampling event for the purpose of modeling, thereby capturing both spatial and temporal variability in occurrence. We fitted a generalized additive model (GAM) with a binomial family and a logit link function to the data [27]. The logit link is the canonical and most widely used link function for binomial data, as it maps the linear predictor to a probability between 0 and 1, and its parameters are readily interpretable as log-odds ratios [28]. The basis dimension (k) for each smooth term in the GAM was conservatively set to 3 [29], providing an upper bound of flexibility (maximum effective degrees of freedom, edf = k − 1) that is appropriate given our sample size, thereby balancing model flexibility with the risk of overfitting. This choice follows the guidance of balancing flexibility and overfitting avoidance. 

To rigorously control for spatiotemporal autocorrelation and prevent inflated performance estimates, we implemented a hierarchical block cross-validation strategy [30]. Temporal validation employed a 4-fold, leave-one-survey-out design, where the model was iteratively trained on data from three surveys and tested on the withheld fourth. Spatial validation used a 5-fold spatial blocking scheme, where all samples from geographically adjacent stations were held out together as a test set. The mean AUC from temporal and spatial cross-validation was 0.96 and 0.97, respectively, confirming that the model’s high predictive performance (final model AUC = 0.98) is robust and not an artifact of autocorrelation. The mean cross-validated AUC is reported as the primary measure of predictive performance.

To control for multicollinearity among predictors, we assessed multicollinearity among predictor variables using variance inflation factors (VIF), removing variables with a VIF greater than 3 [31]. All retained variables were then incorporated into the GAM. Final variable selection was based on the significance of chi-square tests (χ^2^, *p* < 0.05), and we evaluated the model fit using the Akaike Information Criterion (AIC) [32].

The model was conducted using R version 4.2.1. The generalized additive model (GAM) was implemented with the “mgcv” package (v 1.9-4) [16], and model performance was assessed using the “pROC” package (v 1.19.0.1) [27].

### 2.5. Statistical Analysis

The data were analyzed and plotted using Microsoft Excel 2019 (Microsoft, Redmond, WA, USA) and R software, version 4.2.1 (Lucent Technologies, Murray Hill, NJ, USA) statistical analysis software, and the results were presented as mean ± standard error. Differences in environmental factors among the four surveys were tested using one-way analysis of variance (ANOVA) followed by Tukey’s post hoc test, with a significance level of *p* < 0.05. Differences in sediment median grain size (D_50_) between the southeastern and northwestern regions were compared using an independent samples *t*-test. The Welch corrected *t*-test was applied as the assumption of equal variances was violated (Levene’s test, *p* < 0.001).

## 3. Results

### 3.1. Environmental Parameters

The four surveys effectively constituted a natural salinity gradient experiment (Table 1). Mean salinity was significantly lower during the third survey (16.99 ± 0.26‰) than in the other three surveys (*p* < 0.05). Conversely, both average dissolved inorganic nitrogen (73.94 ± 1.17 μmol/L) and chlorophyll-a concentration (13.39 ± 0.89 μg/L) increased significantly during the third survey (*p* < 0.05). Critically, these water column perturbations did not change sediment properties (D_50_ range: 102.59–103.22 μm; *p* > 0.05), thereby establishing the benthic substrate as a stable template that allowed us to isolate salinity effects. The stability of this sedimentary template underscores that the dramatic biological responses we observed arose primarily from water-column perturbations, not from concomitant shifts in the substrate.

### 3.2. Spatial Distribution of Ruditapes philippinarum

**Figure 2:**
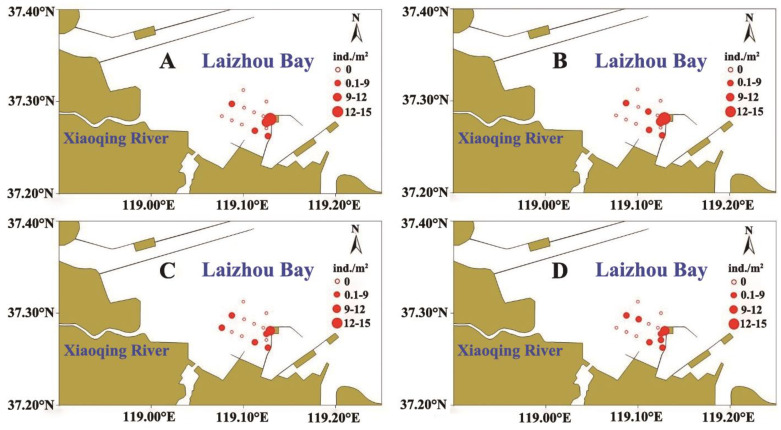
Spatial distribution of *Ruditapes philippinarum* abundance (transformed by log_2_(X + 1) in four surveys in 2024: (**A**) 8 August, (**B**) 22 August, (**C**) 4 September, (**D**) 24 September. The size of the circle indicates the abundance intensity.

The log_2_(X + 1)-transformed abundance of *R. philippinarum* from the four surveys is shown in Figure 2. The third survey (4 September) exhibited a lower abundance compared to the other three surveys. Overall, clams were primarily distributed in the southeastern study area, whereas other regions showed relatively low abundance. This spatial asymmetry presaged the threshold effects: southeastern sediments were significantly finer (D_50_ = 90.89 ± 0.65 μm) than northwestern sediments (D_50_ = 111.93 ± 0.21 μm; t = −30.73, df = 70.08, *p* < 0.001). In terms of size, no significant differences (*p* > 0.05) were observed in average shell length or height among surveys (Table 2).

### 3.3. Optimal Model

The actual edf estimated for each term (Table 3) remained well below this maximum, confirming the chosen complexity was parsimonious and supported by the data. The model was trained using 80% of the data. From eight initial parameters evaluated (T, Sal, DO, Chl-a, DIN, PO_4_-P, D_50_, and SOM), three (D_50_, Sal, and DIN) were retained in the final GAM (Table 3).

The optimal model structure was *Y*~s(D_50_) + s(Sal) + s(DIN), where s( ) denotes a spline smoothing function. This model explained for 79.30% of the deviance and had an AIC value of 46.81.

### 3.4. Evaluation and Validation of the Model

**Figure 3:**
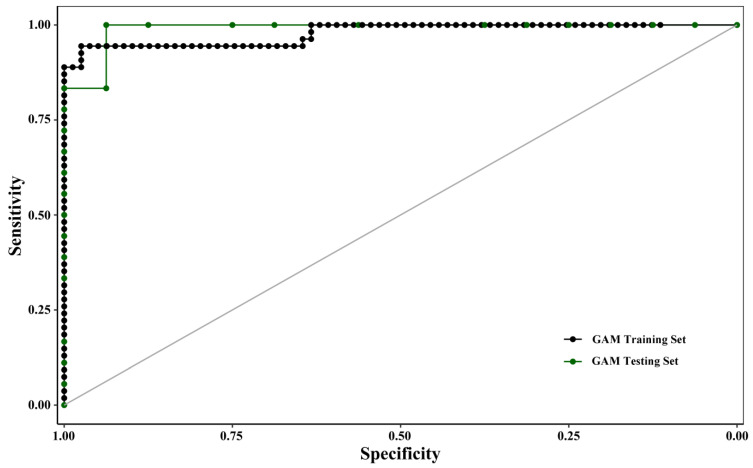
Evaluation results of the GAM model based on ROC curves. Receiver Operating Characteristic (ROC) curves evaluating the predictive performance of the binomial Generalized Additive Model (GAM) for *Ruditapes philippinarum* occurrence. The solid line and dashed line represent the model’s performance on the training set (80% of data) and the independent validation set (20% of data), respectively. The area under the curve (AUC) values (training: > 0.9; validation: > 0.9) demonstrate excellent discriminatory power and near-optimal classification performance.

The evaluation and validation results of the trained GAM are presented in Figure 3 and Table 4. Solid and dashed lines represented the ROC curves for model training and validation, respectively, and the area under these curves (AUC) quantified predictive accuracy. The GAM achieved AUC values exceeding 0.9 for both training and validation, indicating excellent discriminatory power and a near-optimal classification performance.

### 3.5. Factors Influencing the Spatial Distribution of Ruditapes philippinarum

**Figure 4:**
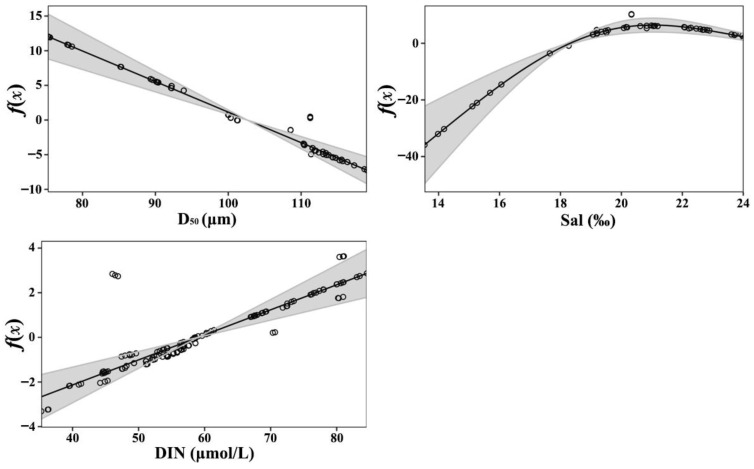
Partial effects of the three key predictors from the binomial Generalized Additive Model (GAM) on the spatial distribution of *Ruditapes philippinarum*. Smooth functions are shown for sediment median grain size (D_50_), salinity (Sal), and dissolved inorganic nitrogen (DIN). The *y*-axis (*f*(*x*)) represents the smooth function on the logit scale. Shaded areas indicate 95% confidence intervals.

The median particle diameter (D_50_) of the sediment was the most significant factor influencing the spatial distribution of *R. philippinarum*, followed by salinity and dissolved inorganic nitrogen (DIN). The GAM results (Figure 4) revealed a negative relationship between species distribution and median particle diameter; as sediment grain size increased, the probability of clam occurrence declined progressively. In contrast, distribution exhibited a positive correlation with both salinity and DIN.

### 3.6. Factors Affecting the Distribution Probability of Ruditapes philippinarum

**Figure 5:**
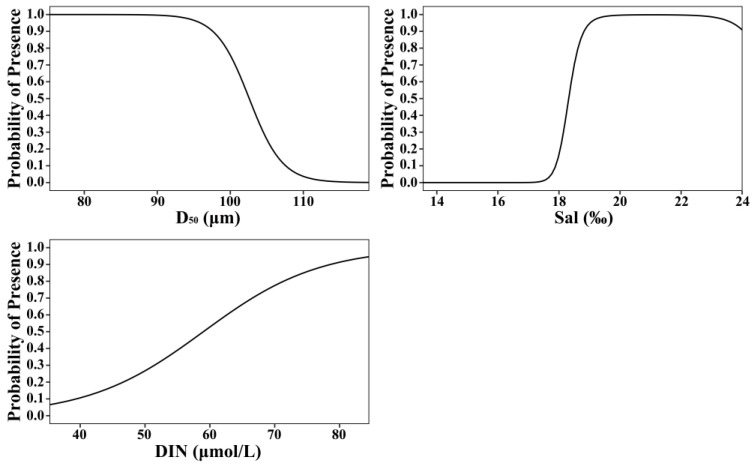
Predicted occurrence probability of *Ruditapes philippinarum* in response to sediment median grain size (D_50_), salinity (Sal), and dissolved inorganic nitrogen (DIN), derived from the binomial Generalized Additive Model (GAM).

In the GAM, the smooth terms for predictors were fitted on the logit scale for the binary response Y. To determine the effect of each factor on the distribution probability of *R. philippinarum*, we back-transformed the smooth functions using the inverse link function. Figure 5 shows the effects of each factor on the spatial distribution probability after back-transformation. The distribution probability of *R. philippinarum* decreased gradually with increasing median grain size, dropping sharply once the median particle size exceeded 95 μm. Within the study area, the distribution probability exhibited a three-stage response to increasing salinity: it remained near-zero at salinities below 17.50‰, increased rapidly between 17.50‰ and 19.50‰, and stabilized above 0.9 once salinity exceeded 19.50‰. As the concentration of dissolved inorganic nitrogen increased, the distribution probability of clams increased, showing an initially rapid rise followed by a gradual slowdown and eventual stabilization.

## 4. Discussion

Our findings resolve the paradox of persistent Manila clam recruitment in Laizhou Bay by demonstrating that microhabitat-scale environmental thresholds override regional degradation. We propose that this persistence is sustained by a refugia network-patches of suitable habitat (D_50_ < 95 μm and Sal > 19.50‰) interconnected by larval dispersal [33] and hydrological processes [5]. Within these patches, substrate suitability buffers against episodic salinity stress, challenging the view that estuarine degradation uniformly compromises ecosystem services. The sharp decline in abundance during the September salinity crisis did not represent a system-wide collapse, as observed in other estuaries following extreme freshwater discharge [34], but rather a spatially selective filter, confining clams to southeastern refugia characterized by persistently finer sediments and higher salinity. The stability of these refugia is likely underpinned by a resilient geomorphic template in which regional sediment transport maintains patches of finer sediments [5] and deeper channels reduce freshwater lensing during runoff events. While our data indicate that refugia are concentrated in the southeastern region with finer sediments, the explicit spatial connectivity and network scale remain to be quantified through hydrodynamic modeling and genetic studies across broader scales.

Within this network, sediment grain size emerges as the primary structural filter for recruitment. The critical threshold at D_50_ = 95 μm is mechanistically grounded in the burrowing biomechanics of bivalves: coarser sediments increase the energetic cost of burrowing, reduce burrow stability, and may limit pedal feeding efficiency [35]. The consistency of this threshold with documented preferences of *R. philippinarum* across globally disparate systems suggests that ~95 μm may represent a species-specific optimum. In our study, clams were present in sediments with a D_50_ ranging from 75.24 to 111.75 μm, with a sharp decline in occurrence probability beyond 95 μm (Figure 5). This range differs from the specific sediment size spectra reported in other systems, such as Sanggou Bay, China (33.60–100.96 μm) and Arcachon Bay, France (50–160 μm) [3,36]. These differences may be attributed to variations in local environmental conditions and population adaptations. Despite these variations, populations across all these regions are consistently found in fine-sand sediments. Consequently, maintaining D_50_ below this threshold through strategic substrate management offers a universal and quantifiable target for enhancing settlement success in restoration initiatives. The temporal stability of the sedimentary template throughout our surveys is notable. Despite significant fluctuations in water-column properties, mean median sediment grain size (D_50_) showed no significant differences across surveys (Table 1; *p* > 0.05), indicating that the benthic substrate acted as a stable habitat matrix. This stability aligns with regional sediment transport patterns in the Xiaoqing River estuary [5] and explains the clear spatial segregation of clams in the southeastern refugia with persistently finer sediments. Crucially, this stable substrate envelope allowed us to isolate the effects of episodic osmotic stress, revealing salinity as the primary dynamic determinant of recruitment success.

Our field surveys captured a natural salinity-stress event (minimum 16.99‰) that triggered system-wide collapse in clam abundance. The GAM attributes this response to a critical threshold at 17.50‰—below which occurrence probability approaches zero and above which probability recovers exponentially up to 19.50‰, delineating a critical “transitional recovery zone” (Figure 5). This finding is mechanistically supported by laboratory studies of osmoregulatory dysfunction [37], specifically the inhibition of Na^+^/K^+^-ATPase activity and activation of transcriptomic stress pathways. More broadly, acute salinity fluctuations induce osmotic stress in bivalves, thereby disrupting physiological processes and leading to mortality [38]. Analogous catastrophic mortality in other bivalves (e.g., *Crassostrea* [34], *Mytilus* [39]) underscores the general threat of acute freshwater pulses. However, direct comparisons of numeric thresholds are complicated by significant interspecific variation in life history and physiological tolerance [40]. Consequently, the 17.50‰ threshold identified here, though derived from a single episodic event, provides a critical preliminary reference point specific to *R. philippinarum* that should not be directly extrapolated to other bivalves without validation. Within the transitional recovery zone, interventions such as temporary water diversion or supplemental aeration could mitigate mass mortality. Consequently, monitoring for salinity declines below 17.50‰ offers a potential, empirically informed early-warning signal that warrants further testing. Before managers can widely apply this threshold to active management of wild and cultured clam populations, they must verify its universality and seasonal stability, requiring testing across multiple years, different estuaries, and various climatic conditions.

The significant increase in DIN during the low-salinity event had only a marginal direct effect on clam distribution, explaining merely 3.54% of deviance (Table 3). More critically, our model shows that acute salinity stress (<17.50‰) reduces distribution probability to near zero (Figure 5), and may completely override any potential DIN benefit. Although Du et al. [34] did not assess nutrient conditions, their study underscores that acute salinity stress alone can drive mass mortality, indicating that elevated nutrients cannot offset the associated physiological collapse. Our findings therefore reveal a fundamental management trade-off: chronic nutrient enrichment may offer limited benefits, but it cannot compensate for lethal salinity or unsuitable sediment. Consequently, restoration efforts must adhere to a hierarchical prioritization: managers should first select sites with suitable substrate (D_50_ < 95 μm), then ensure salinity stability (>19.50‰) (Figure 5), and only thereafter consider the secondary effects of DIN. Attempting to offset poor habitat quality through fertilization is ecologically unsound and practically ineffective.

Building upon these thresholds identified in the Xiaoqing River estuary, and acknowledging the need for further validation for broader application, these results define a non-negotiable management hierarchy: sediment grain size (D_50_ < 95 μm) and salinity avoidance (Sal < 17.50‰) (Figure 5) serve as primary, non-compensatory controls, whereas the positive effect of DIN is secondary. To operationalize these thresholds, we propose the following actionable framework in Laizhou Bay:

1. Habitat-suitability mapping for restoration: The D_50_ < 95 μm threshold provides a clear, measurable criterion for spatially prioritizing restoration efforts. We recommend developing habitat-suitability maps from routine sediment surveys to direct resources exclusively to patches that meet this foundational substrate condition.

2. Tiered early-warning system and policy integration for salinity risk: The Sal = 17.50‰ threshold should serve a dual role: as the core of a real-time, tiered alert system and as a key benchmark integrated into watershed management policies. Operationally, monitoring networks should trigger a ‘watch’ when salinity approaches this level, an ‘action’ alert upon sustained breach (prompting managers to implement immediate interventions such as adjusting managed water flows in critical habitats), and an ‘emergency’ response during prolonged exposure to prevent mass mortality. In parallel, water-management policies should institutionalize this threshold, specifically to regulate freshwater-discharge volumes during critical biological windows (e.g., spawning and recruitment seasons). This dual approach ensures both rapid reactive protection and long-term proactive mitigation of anthropogenic salinity stress, thereby safeguarding nursery habitats and stock resilience.

3. Clarified restoration prioritization: Our framework establishes a clear decision sequence: substrate first, salinity second, nutrients third. This hierarchy ensures that managers allocate management and restoration resources efficiently, preventing the ecologically unsound practice of attempting to fertilize unsuitable habitats.

This framework is presented as a prototype developed from the Xiaoqing River estuary case. Its core principles (substrate-salinity hierarchy) are likely transferable to similar temperate estuaries, while specific thresholds (especially for salinity) should be locally calibrated and validated across seasons and years. This approach facilitates a shift from generic management to proactive, science-based stewardship tailored to specific ecosystems.

We acknowledge that using adult presence as a proxy for recruitment potential has inherent limitations: Adult distribution integrates cumulative outcomes of multiple life-history stages (larval settlement, post-settlement survival, adult persistence), and thus cannot distinguish between high-settlement/high-mortality and low-settlement/high-survival scenarios, nor directly quantify thresholds for early-life stages. Adult distributions may also reflect predation pressure, fishing, or migration, pressures not assessed here. Importantly, the sharp, non-linear thresholds we identified for sediment (D_50_ > 95 μm) and salinity (<17.50‰) align with known physiological and biomechanical constraints for *R. philippinarum*, lending mechanistic credibility to our field-derived models. We therefore interpret these thresholds as the limits of habitat suitability that support persistent adult populations—a robust indicator of a site’s capacity for sustained natural recruitment under fluctuating environmental conditions. Future studies that incorporate spat monitoring, larval-dispersal modeling, and controlled experiments across salinity and sediment gradients would further refine our understanding of recruitment bottlenecks and validate these thresholds for early-life stages.

## 5. Conclusions

This study identifies two critical, data-driven thresholds that provide the scientific basis for managing Manila clam populations in degraded estuaries. First, our GAM results establish a sediment filter (D_50_ < 95 μm), which can inform the scientific prioritization of habitat restoration sites. Second, the model identifies a salinity threshold (17.50‰) that serves as an empirical benchmark for implementing early-warning systems against climate- and human-induced mortality risks. Third, the quantified hierarchy of predictor effects (D_50_ > Sal > DIN) underscores that substrate suitability and salinity are primary and non-compensatory controls, whereas the positive effect of DIN is secondary and cannot offset stress from poor sediment or low salinity. These field-validated thresholds and their relationships enable a strategic shift from passive observation to predictive, resilience-based management of *R. philippinarum* and potentially other temperate estuarine bivalves facing similar stressors.

## Figures and Tables

**Table 1:** Environmental characteristics (mean ± SE) of water and sediment at the sampling sites in the Xiaoqing River estuary across four surveys in 2024.

	Parameters	8 August	22 August	4 September	24 September
Water	T (°C)	30.03 ± 0.02 ^a^	30.33 ± 0.04 ^b^	26.06 ± 0.04 ^c^	21.13 ± 0.10 ^d^
	Sal (‰)	21.92 ± 0.26 ^c^	20.79 ± 0.22 ^b^	16.99 ± 0.26 ^a^	21.35 ± 0.21 ^bc^
	DO (mg/L)	3.45 ± 0.08 ^b^	3.05 ± 0.08 ^a^	4.98 ± 0.06 ^c^	5.52 ± 0.05 ^d^
	DIN (μmol/L)	51.15 ± 0.86 ^a^	50.17 ± 1.01 ^a^	73.94 ± 1.17 ^c^	62.79 ± 0.94 ^b^
	PO_4_-P (μmol/L)	8.23 ± 0.80 ^a^	9.56 ± 0.54 ^a^	9.91 ± 0.59 ^a^	16.32 ± 0.57 ^b^
	Chl-a (μg/L)	5.86 ± 0.17 ^ab^	6.51 ± 0.41 ^b^	13.39 ± 0.89 ^c^	4.69 ± 0.16 ^a^
Sediment	D_50_ (μm)	103.22 ± 2.09 ^a^	102.59 ± 2.08 ^a^	102.90 ± 2.07 ^a^	103.03 ± 2.09 ^a^
	SOM (%)	1.83 ± 0.20 ^b^	1.21 ± 0.09 ^a^	1.47 ± 0.09 ^a^	1.35 ± 0.08 ^a^

Notes: Data labeled with different letters are significantly (*p* < 0.05) different from each other within each row. Different lowercase superscript letters (a, b, c, d) within a row indicate statistically significant differences (*p* < 0.05) among sampling dates, as determined by one-way ANOVA followed by Tukey’s post hoc test. Abbreviations: T, temperature; Sal, salinity; DO, dissolved oxygen; DIN, dissolved inorganic nitrogen; PO_4_-P, phosphate; Chl-a, chlorophyll-a; D_50_, median sediment grain size; SOM, sediment organic matter.

**Table 2:** Shell length and height of *Ruditapes philippinarum* from four surveys. Shell length and height of *Ruditapes philippinarum* collected during the four surveys in the Xiaoqing River estuary, 2024.

Survey Date	Shell Length (mm)	Shell Height (mm)
8 August	19.64 ± 0.51	12.86 ± 0.39
22 August	18.45 ± 0.26	12.03 ± 0.18
4 September	18.35 ± 0.24	12.82 ± 0.11
24 September	18.25 ± 0.23	11.88 ± 0.17

Notes: Values are presented as mean ± standard error (SE). No significant differences (*p* > 0.05) were found in either shell length or height among the four survey dates based on one-way ANOVA.

**Table 3:** Summary of the optimal binomial Generalized Additive Model (GAM) predicting the occurrence of *Ruditapes philippinarum*.

Parameters	edf	*p*	Cumulative Deviance Explained (%)	Deviance Explained (%)	AIC
D_50_	1	<0.001	60.65	60.65	46.81
Sal	1.86	0.007	75.80	15.15	
DIN	1	0.030	79.34	3.54	

Notes: edf, estimated degrees of freedom (reflecting the non-linearity of the smooth term); AIC, Akaike Information Criterion (lower values indicate better model fit). Predictors are listed in descending order of individual contribution to deviance explained.

**Table 4:** Performance metrics of the optimal binomial GAM for *Ruditapes philippinarum* occurrence during model training and independent validation.

Model Evaluation or Validation	*μY* Threshold	Sensitivity	Specificity	AUC
model evaluation	0.36	0.94	0.97	0.99
model validation	0.29	1	0.94	0.98

Notes: The model was trained on a randomly selected 80% subset of the data (*n* = 168) and validated on the remaining 20%. AUC, area under the Receiver Operating Characteristic curve. An AUC value of 1 represents perfect discrimination, while 0.5 represents no discriminative ability. The high AUC values (>0.98) in both stages indicate excellent model performance and strong predictive power.

## Data Availability

Data will be made available on request.
